# Positively Correlated CD47 Activation and Autophagy in Umbilical Cord Blood-Derived Mesenchymal Stem Cells during Senescence

**DOI:** 10.1155/2021/5582792

**Published:** 2021-04-15

**Authors:** Gee-Hye Kim, Yun Kyung Bae, Ji Hye Kwon, Miyeon Kim, Soo Jin Choi, Wonil Oh, Soyoun Um, Hye Jin Jin

**Affiliations:** Biomedical Research Institute, MEDIPOST Co., Ltd., Seongnam 13494, Republic of Korea

## Abstract

Autophagy plays a critical role in stem cell maintenance and is related to cell growth and cellular senescence. It is important to find a quality-control marker for predicting senescent cells. This study verified that CD47 could be a candidate to select efficient mesenchymal stem cells (MSCs) to enhance the therapeutic effects of stem cell therapy by analyzing the antibody surface array. CD47 expression was significantly decreased during the expansion of MSCs in vitro (*p* < 0.01), with decreased CD47 expression correlated with accelerated senescence phenotype, which affected cell growth. UCB-MSCs transfected with CD47 siRNA significantly triggered the downregulation of pRB and upregulation of pp38, which are senescence-related markers. Additionally, autophagy-related markers, ATG5, ATG12, Beclin1, and LC3B, revealed significant downregulation with CD47 siRNA transfection. Furthermore, autophagy flux following treatment with an autophagy inducer, rapamycin, has shown that CD47 is a key player in autophagy and senescence to maintain and regulate the growth of MSCs, suggesting that CD47 may be a critical key marker for the selection of effective stem cells in cell therapy.

## 1. Introduction

Autophagy is a natural intracellular degradation mechanism that maintains cellular homeostasis by delivery to the lysosome [1]. The coordinated response to starvation and metabolic stress triggers autophagy to function in the recruitment and degradation of cytosolic proteins and organelles to remove and recycle any malfunctioning or unnecessary components [2]. In pathological conditions, including neurodegenerative diseases, cancer, and inflammatory diseases, autophagy is also induced and modulated. It is clear that autophagy plays a role in controlling inflammation and is closely implicated in human disease [1, 3, 4]. Autophagy, an intracellular self-degradation system, is responsible for the removal of damaged organelles or malformed proteins, in order to regenerate newer and healthier cells. The function of autophagy is related to cellular clearance, which is critical for development, differentiation, and tissue remodeling [5–8]. Therefore, understanding autophagy function is important to clarify the cause of diseases. Autophagy has a bifunctional role in cell survival and death, presumably by clearing potential toxic protein aggregates. Blocking autophagy suppresses cytochrome c release from mitochondria and caspase activation and induces cell viability [9].

Aging, affecting the decline in regenerative potential of stem cells, is coupled with a progressive reduction in the regulation of cellular and tissue interactions. It results in senescence, allowing irreversible arrest of cellular division. Insufficient clearance of accumulation in damaged components is mainly described in aging organisms. An alternating lysosomal degradation system and decreased autophagic activity were ensued by aging. Commonly, impaired and defective autophagy in aged cells has been identified [10, 11]. The decline in both the number and functionality of stem cells with aging may contribute to regenerative decline. It has been reported that autophagy is related to age-dependent changes in stem cells for maintenance of stemness and differentiation capacity [12]. Additionally, immunosuppressive properties of higher passage mesenchymal stem cells (MSCs) are decreased, notwithstanding the stable phenotype of MSCs [13, 14]. There was a significant decline in self-renewal capacity with increased donor age and in vitro expansion [15–17]. Interestingly, higher passaged cells and cells from old donors showed less proliferative capacity. In addition, secretion of cytokines and growth factors from less proliferative cells was related to senescence-associated secretory phenotype, which is associated with senescence [18, 19]. Therefore, it is important to find key modulatory mechanisms of senescence markers to determine the quality of the in vitro cells for stem cell therapy.

CD47, known as integrin-associated protein (IAP), is a cell surface glycoprotein expressed in human cells and binds the ligands thrombospondin-1 (TSP-1) and signal-regulatory protein alpha (SIRP*α*) [20]. Clinically, the inhibition of CD47 is a potential therapeutic strategy for treatment of various cancers. The absence of CD47 results in phagocytosis of T-cell-mediated adaptive immunity by targeting CTLA and PD-1 [21]. Recent studies have focused on overexpressed CD47, which plays a key role in immune response in tumor cells [22–24].

In addition to immune responses, CD47 is involved in a range of cellular processes, including apoptosis, proliferation, adhesion, and migration. Activation of CD47 was correlated with enhanced proliferation of cancerous cells via the P13K/Akt pathway [25]. On the other hand, activation of CD47 with TSP-1 inhibited proliferation and suppressed expression of stem cell transcription factors, such as Sox2, Klf4, and Oct4 [26]. CD47 also leads to cell death in normal and tumor cells via apoptosis or autophagy. However, the relationship between CD47 and autophagy or senescence had a functional significance, which was unclear at this time. This study reported that CD47 is a key regulator of autophagy and senescence to maintain and orchestrate the aging of MSCs.

## 2. Materials and Methods

### 2.1. Cell Culture

The Institutional Review Board of MEDIPOST Co., Ltd. approved this study (MP-2014-07-1-1). To culture UCB-MSCs, UCB was collected in blood bags containing citrate phosphate dextrose adenine (CPDA-1), anticoagulant, within 24 h from mothers who gave informed consent. Human mononuclear cells (MNCs) were isolated from UCB with Ficoll-Hypaque solution (Sigma-Aldrich, St. Louis, MO, USA). The isolated MNCs were suspended in minimum essential medium *α* (Gibco, Carlsbad, Grand Island, NY, USA) supplemented with 10% fetal bovine serum (FBS; Gibco) and gentamicin (Gibco) at 37°C in a 5% 05CO_2_ incubator. Medium was changed every three days. Nonadherent cells were washed out with medium changes. Adherent cells, MSCs, were subcultured at 70% confluence. Cell expansion was analyzed using the trypan blue exclusion method. Rapamycin (10 *μ*M, Sigma-Aldrich), an inducer of autophagy, was also treated with UCB-MSCs (passage 6, P6) for 3 h.

### 2.2. Flow Cytometry and Cell Surface Antibody Screening with Lysoplates

To detect CD47 and stem cell markers of MSCs (P5), fluorescence-activated cell sorting was performed. Cells were detached and washed with DPBS and then incubated with fluorescein isothiocyanate- (FITC-) conjugated human CD47, CD14, CD45, CD146, and human leukocyte antigen- (HLA-) DR antibodies (BD Biosciences, San Diego, CA, USA) and phycoerythrin- (PE-) conjugated human CD49b, CD73, CD166, CD274, epidermal growth factor receptor (EGFR; BD Biosciences), CD90, and CD105 antibodies (Invitrogen, Carlsbad, CA, USA) for 20 min. An isotype control was also included [27]. Cells were washed with DPBS and fixed with 4% paraformaldehyde. MSC immunophenotypes were determined by flow cytometry on a MACS Quant flow cytometer (Miltenyi Biotec, Bergisch Gladbach, Germany), and expression of cell surface antigens was calculated for 10,000 gated-cell events. For screening cell surface markers, 5 × 10^5^ cells isolated from two groups (Groups 1 and 2) were dispensed into 96-well round-bottom plates (BD Lysoplates, BD Biosciences), which were lyophilized with 242 human cell surface marker antibodies. Stained cells were washed and subsequently stained with an Alexa Fluor 647-conjugated goat-anti-mouse IgG secondary antibody (Thermo Fisher Scientific, Eugene, OR, USA). Surface markers were measured by flow cytometry on a FACSCalibur instrument (BD Biosciences) by calculating the percentage of cells per 10,000 cell events.

### 2.3. Human Autophagy Array

Autophagy-related protein levels were analyzed by Human Autophagy Array C1 (RayBiotech, Norcross, GA, USA) using 20 different antibodies. Briefly, 2 mg cell lysates were incubated with an antibody array membrane at 4°C. The day after, repeated washings with the provided buffer were performed. Each membrane was incubated with biotinylated antibody cocktail for 2 h, followed by treatment of HRP-Streptavidin for 2 h. After further washing, membranes were incubated with detection buffer and detected on a ChemiDoc Imaging System (Bio-Rad, Hercules, CA, USA). The intensity of the protein array was normalized against positive control spots.

### 2.4. SA *β*-Gal Staining

Cellular senescence was analyzed using a Senescence *β*-Galactosidase Staining Kit (Cell Signaling Technology, MA, USA). UCB-MSCs at passages 4, 7, and 13 were 70% confluent. Cells were fixed with fixing solution for 5 min at RT. After washing twice with DPBS, cells were incubated with staining working solution for 48 h at 37°C in darkness. Cells were examined using an inverted microscope (Nikon, Japan). The average percentage of stained cells was calculated from four fields.

### 2.5. Western Blotting

Cells were lysed with radioimmunoprecipitation assay buffer (Thermo Fisher Scientific, Waltham, MA). A total of 15 *μ*g of each protein extract was electrophoresed on a sodium dodecyl sulfate-polyacrylamide gel and then transferred to a nitrocellulose membrane. Blocked membranes were incubated with primary antibodies (anti-human Beclin1, anti-human Atg5, anti-human Atg12, anti-human phospho-p38 MAPK (Thr190/Tyr182, pp38), and phospho-Rb (Ser780, pRB, Cell Signaling, Danvers, MA, USA); anti-human LC3B (Sigma-Aldrich); anti-human CD47 (Abcam, Cambridge, MA, USA); and anti-human GAPDH (Novus bio, Centennial, CO, USA)) overnight at 4°C and then probed with horseradish peroxidase-conjugated secondary antibodies for 1 h at room temperature. A chemiluminescence immunoblotting system (GE Healthcare Life Sciences, Buckinghamshire, UK) was used to visualize and analyze the bands.

### 2.6. Small Interfering RNA (siRNA)

Cells were transfected with CD47 small interfering RNAs (siRNAs) and scrambled siRNA (100 nM, Dharmacon, Lafayette, CO, USA) for 48 h using the DharmaFECT reagent according to the manufacturer's recommendations. Human CD47 siRNA with sequences 5′-GCAUGGCCCUCUUCUGAUU-3′, 5′-GUACAGCGAUUGGAUUAAC-3′, 5′-CAGAGAAGGUGAAACGAUC-3′, and 5′-UAACUGAAGUGAAGUGAUG-3′ was synthesized. Scrambled siRNA with sequences 5′-UGGUUUACAUGUCGACUAA-3′, 5′-UGGUUUACAUGUUGUGUGA-3′, 5′-UGGUUUACAUGUUUUCUGA, and UGGUUUACAUGUUUUCCUA-3′ was synthesized.

### 2.7. Immunocytochemistry

To detect CD47 and LC3B, cells were seeded on two-chamber slides. After washing with PBS, cells were fixed with 4% PFA and then blocked with 0.1% Triton X-100. Cells were incubated with CD47 primary antibodies (1 : 500, Abcam) and secondary antibody (anti-mouse Cy3-conjugated secondary antibody, 1 : 700, Jackson ImmunoResearch Europe Ltd., Newmarket, UK). LC3B was detected using a rabbit monoclonal primary antibody (1 : 1000, Abcam) followed by an anti-rabbit Alexa Fluor® 488 (1 : 700, ImmunoResearch Europe Ltd). Images were acquired using an LSM 800 confocal microscope (Zeiss, Oberkochen, Germany).

### 2.8. Statistical Analysis

All data is presented as the mean ± standard deviation. Statistical analysis was performed with a one-way analysis of variance (ANOVA) for experiments with more than two groups followed by Tukey's HSD test using GraphPad Prism (San Diego, CA, USA). All experiments were repeated at least three times. Data was considered statistically significant if *p* < 0.05.

## 3. Results

### 3.1. CD47 Affects Cellular Processes on UCB-MSCs

To confirm that cell surface protein on senescent MSCs controls the aging process, 242 human cell surface antibody screening was performed. Our previous study found that melanoma cell adhesion molecule (MCAM/CD146) among 242 human cell surface markers was downregulated with prolonged in vitro expansion, associated with cellular senescence [26]. In the screening results of senescent UCB-MSCs, cell surface markers, CD47, CD146, CD49b, CD274, and EGFR, which showed a significant difference between early and late passages, were selectively chosen. Proliferative rates on UCB-MSCs (P5) under the same conditions were measured in 10 different MSC lots to verify the effects of senescent states. The basic characteristics of the MSCs, such as stemness, are determined with stem cell surface markers (Table S1). MSCs were positive to CD90, CD73, CD166, and CD105 (≥85%). CD14, CD45, and HLA-DR MSC markers were negative (≤1%). The differentiation capacity is determined by ALPase staining and Von Kossa staining.

**Figure 1:**
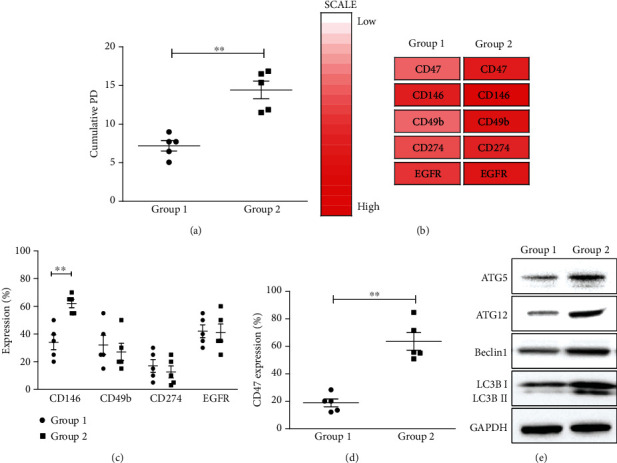
Screening results revealed different CD47 expressions on two groups, depending on cellular proliferation. (a) Comparative results of cumulative population doubling (CPD) at passage 5 showed higher proliferative capacity on Group 2. (b) Screening for cell surface proteins between two groups was shown with heat map analysis based on 242 cell surface antibody screening. The significant difference in cell surface antigen expression between two groups was revealed: CD47, CD146, CD49b, CD274, and EGFR. (c, d) Flow cytometry identified two CD markers (CD146 and CD47), showing significant difference expression. (e) Immunoblotting results demonstrated that the expression of autophagy-related proteins, ATG5, ATG12, Beclin1, and LC3B, downregulated on Group 1. Data is expressed as the mean ± SD; *n* = 5; ^∗∗^*p* < 0.01, ^∗^*p* < 0.05.

Depending on the proliferative rate, UCB-MSC lots were divided into two groups (Group 1 and Group 2). Group 2 had a higher cumulative population doubling (PD) at passage 5, compared to that of Group 1 (Figure 1(a)). Heat map analysis showed downregulated surface proteins (CD47, CD146, CD49b, CD274, and EGFR) in Group 1 (Figure 1(b)). Flow cytometric analysis revealed that only the CD146 and CD47 protein expressions were significantly suppressed in Group 1, with less proliferative capacity. There was a significant difference in CD47 levels between Group 1 and Group 2 (Figures 1(c) and 1(d)). To evaluate the cellular processes between senescence and autophagy, immunoblotting was performed with autophagy-related proteins, ATG5, ATG12, Beclin1, and LC3B in Groups 1 and 2 (Figure 1(e)). At the same time, Group 1 and Group 2 showed significant differences in autophagy-related proteins. The increased expression of ATG5, ATG12, Beclin1, and LC3B was analyzed, suggesting that senescent cells may regulate autophagic activity during passages.

### 3.2. The Relationship between CD47 and Cellular Senescence in the Late Passage UCB-MSCs

**Figure 2:**
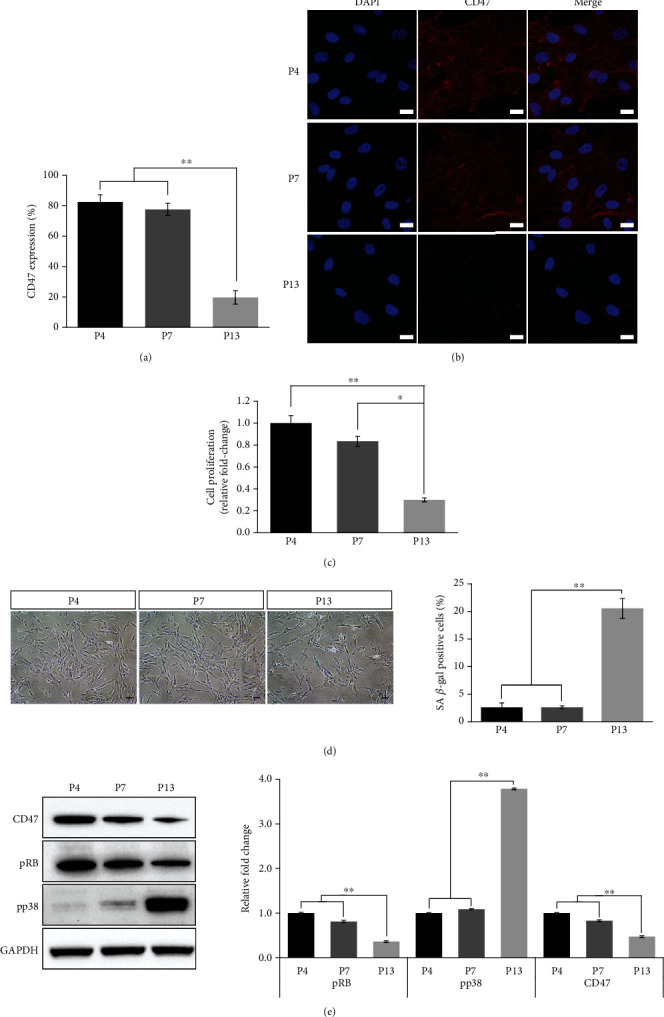
CD47 expressions and cellular senescence on late passages of UCB-MSCs were downregulated. (a) Flow cytometric analysis of CD47 expressions on UCB-MSCs during passaging was performed. (b) Representative immunofluorescence images of CD47-positive cells under passaging from passage 4 to 13. Scale bar 20 *μ*m. (c) Cell proliferation was determined by measuring fold changes, with results normalized to the growth observed at passage 4. (d) SA *β*-gal staining was performed on UCB-MSCs at passages 4, 7, and 13. SA *β*-gal positive cells were quantified by determining the percentages of stained cells. Scale bar 50 *μ*m. (e) Representative western blots of CD47, pRB, and pp38, cellular senescence-related markers, in UCB-MSCs subjected to passaging the cells. Semiquantification of protein expression levels of CD47, pRB, and pp38 was analyzed at passage 4 (P4), passage 7 (P7), and passage 13 (P13). Data is expressed as the mean ± SD; *n* = 3; ^∗∗^*p* < 0.01, ^∗^*p* < 0.05.

To evaluate the relationship between cellular senescence and cell surface marker CD47, CD47 expression was analyzed in Group 2 during passage from P4 to P13. Until P7, there was no significant change in CD47 expression in MSCs. However, CD47 expression was significantly decreased at a late passage, P13 (Figure 2(a)). Representative confocal microscopic images of CD47-positive cells at a late passage (P13) showed significantly decreased CD47 expression (*p* < 0.01, Figure 2(b)). Overall, passaging of MSCs downregulated cell surface CD47 expression. The fold changes in cell growth gradually decreased from P4 to P13 (Figure 2(c)). To clarify the general cellular senescence processes, SA *β*-gal activity was monitored during passaging from P4 or P7, and P13. SA *β*-gal positive cells gradually increased from P4 to P13. At P13, cellular senescent activity was significantly increased compared to early passages (P4 and P7) (Figure 2(d)). Moreover, western blotting showed decreased CD47 protein levels from P4 to P13. The level of pRB, a senescence-related protein, was significantly decreased at P13. Conversely, the level of pp38, a senescence-related protein, was significantly increased at P13 (*p* < 0.01, Figure 2(e)). Overall, the results demonstrated that UCB-MSC expansion increased senescent progression, consistent with downregulated CD47 expression.

### 3.3. The Relationship between CD47 and Autophagy in the Late Passage UCB-MSCs

**Figure 3:**
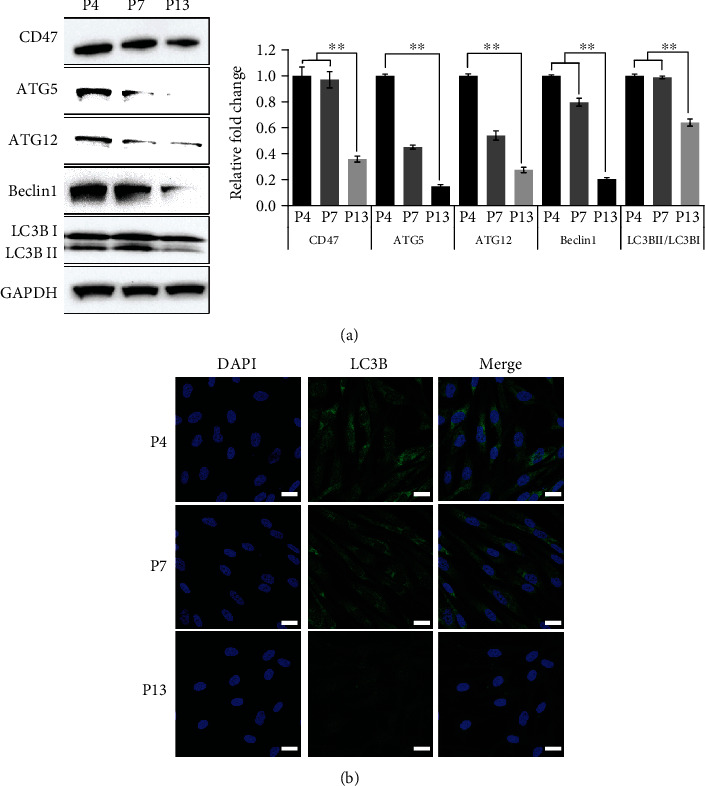
CD47 expressions and autophagy on UCB-MSCs were downregulated during passages. (a) Representative western blot results revealed autophagy-related protein expressions, CD47, ATG5, ATG12, Beclin1, and LC3B during passaging from P4 to P13. Relative fold changes of protein expressions were normalized to each protein level observed at P4. (b) LC3B-positive cells at different passages (P4, P7, and P13) were monitored by immunostaining. Scale bar 20 *μ*m. Data is expressed as the mean ± SD; *n* = 3; ^∗∗^*p* < 0.01.

The correlation between CD47 and autophagy phenotype at late passages of UCB-MSCs was confirmed by immunoblotting of autophagy-related markers and fluorescent staining of LC3B. To evaluate autophagy during passaging from P4 to P13 of UCB-MSCs, ATG5, ATG12, Beclin1, and LC3B expression levels on UCB-MSCs were markedly decreased at P13 (Figure 3(a)). Confocal microscopy images demonstrated markedly decreased density of LC3B-positive cells, an autophagosome marker, in the late passage (Figure 3(b)). The result was consistent with downregulated protein levels of autophagy-related markers in UCB-MSCs. These results suggest that the senescent UCB-MSCs have lower autophagy, which can be correlated by the lower CD47 expression.

### 3.4. CD47 Knockdown Reduces Autophagy and Accelerates the Senescence of UCB-MSCs

**Figure 4:**
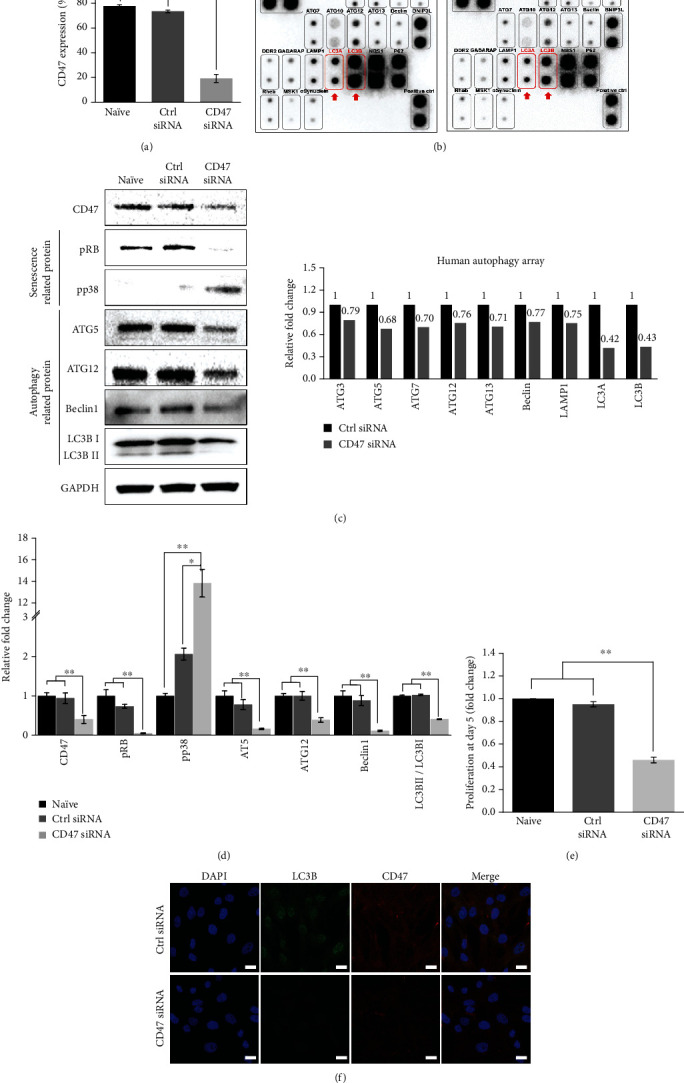
CD47 knockdown in UCB-MSCs reduces the autophagy and accelerates the senescence. (a) Flow cytometric analysis showed CD47-silenced UCB-MSCs downregulated CD47 cell surface markers at passage 6. (b) Human autophagy array was performed to reveal the significant autophagy-related protein level difference between control and CD47-silenced UCB-MSCs. Relative fold changes were normalized to control siRNA-transfected cells. Red box with arrow indicated LC3B proteins, showing significant difference. (c, d) Representative western blot analysis demonstrated relative changes of senescence-related proteins (pRB and pp38) and autophagy-related proteins (ATG5, ATG12. Beclin1, and LC3B) after CD47 siRNA transfection at passage 6. (e) Proliferation at day 5 after CD47 siRNA transfection was downregulated. (f) Immunostaining results with LC3B and CD47 antibodies on control siRNA and CD47 siRNA-transfected UCB-MSCs were determined. Scale bar 20 *μ*m. Data is expressed as mean ± SD; *n* = 3; ^∗∗^*p* < 0.01.

To investigate the role of CD47 in autophagy and senescence, the cellular profile of CD47 knockdown UCB-MSCs and normal UCB-MSCs was assessed. CD47, a cell surface marker at passage 6, was silenced. MSCs were transfected with 100 nM CD47 siRNA or a scrambled control siRNA for 48 h. CD47 siRNA treatment significantly suppressed CD47 protein levels, as shown by flow cytometry results (Figure 4(a)).

Human autophagy array results showed significant differences in LC3A and LC3B protein levels between scrambled control siRNA-transfected and CD47 siRNA-transfected MSCs. The 60% protein levels of LC3A and LC3B, autophagy-related markers, were downregulated in CD47 siRNA-treated MSCs. In addition to LC3A and LC3B, ATG3, ATG5, ATG7, ATG13, Beclin, and LAMP1, autophagy-related proteins, revealed 20% suppression of protein levels with CD47 blockade. As depicted in Figures 4(b) and 4(c), the marked changes in LC3A and LC3B after CD47 siRNA treatment correlated with the alteration of CD47 protein levels. Overall, CD47 knockdown in UCB-MSCs reduced autophagy via LC3A and LC3B.

Interestingly, western blot results demonstrated that pRB and pp38 levels, senescence-related proteins, were also affected by CD47 knockdown. With CD47 siRNA transfection, pRB proteins were inhibited, whereas the level of pp38 proteins revealed a 14-fold increase in CD47 siRNA-transfected MSCs (Figure 4(c)). Relative fold changes of CD47, pRB, pp38, ATG5, ATG12, Beclin1, and LC3B were analyzed with significant differences between scrambled control siRNA and CD47 siRNA-transfected MSCs (Figure 4(d)). To verify the role of CD47 on proliferation, CD47-transfected MSC counts were demonstrated during day 5. Downregulated CD47 expression also inhibited UCB-MSC proliferation (Figure 4(e)). Representative immunofluorescent staining has been implicated in the relationship between LC3B and CD47. LC3B-positive cells were significantly reduced, correlated with CD47 protein levels, with CD47 siRNA transfection (Figure 4(f)). Thus, CD47 may affect LC3B expression that accumulates autophagy and triggers senescence in late passages of UCB-MSCs.

### 3.5. CD47 Influences Senescence in UCB-MSCs

**Figure 5:**
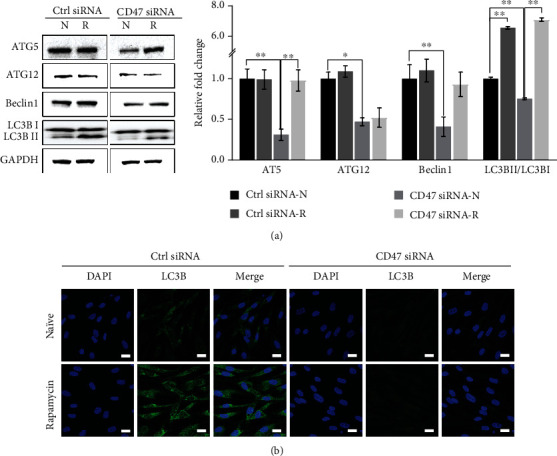
Autophagy flux. (a) Rapamycin (R, 10 *μ*M), autophagy inducer, was treated in CD47 knockdown UCB-MSCs. Autophagy-related proteins were determined with immunoblotting analysis. Relative fold change of proteins, ATG5, ATG12, Beclin1, and LC3BII relative to LC3BI was quantified against protein levels of GAPDH. (b) Representative immunofluorescence images of LC3 with treatment of autophagy inducer or inhibitors showed significant different intensities on LC3 levels, contingent upon CD47 levels. Scale bar 20 *μ*m. Data is expressed as the mean ± SD; *n* = 3; ^∗∗^*p* < 0.01, ^∗^*p* < 0.05.

Autophagic flux in CD47 siRNA-transfected UCB-MSCs is estimated by treatment of the inducer, rapamycin. Autophagy induction during passaging on MSCs converts LC3BI to LC3II and induces an increase in LC3B. Rapamycin, an inducer of autophagy, inhibits the mTOR signaling pathway, which downregulates autophagy indirectly by negatively regulating the transcription of genes required for lysosomal function. Rapamycin upregulated LC3BII expression by inducing autophagy in the scrambled control group (Figure 5(a)). However, immunoblotting results demonstrated that Beclin 1 and LC3B-II levels downregulated in CD47 siRNA transfected group were enhanced by rapamycin treatment. To measure autophagic flux, it is essential to determine the extent to which LC3BII is degraded in a lysosome-dependent manner and how much LC3 puncta are determined by fluorescent staining. The increase in the number of LC3 puncta of naïve cells in the scrambled control group in the presence of rapamycin represented the number of autophagosomes. LC3B was recruited to autophagosomes forming punctate structures, as indicated by the green color. However, CD47-transfected groups showed a decreased number of LC3B-positive puncta in the presence of rapamycin (Figure 5(b)). Together, these results establish that CD47 is critical for modulating autophagic flux via LC3B-related lysosome regulation.

## 4. Discussion

This study demonstrated CD47 as a critical mediator of proliferation and autophagy to maintain and control MSC senescence. Based on various beneficial functions, including stemness, differentiation potential, paracrine action, low immunogenicity, and tumorigenicity, MSCs have been widely applied in cell-based trials for broader-spectrum diseases. To obtain a sufficient number of cells for sufficient therapeutic effect, MSCs must be expanded during long-term in vitro culture, followed by premature senescence. Cellular senescence potentially induces poor clinical outcomes by producing growth arrest and reducing stem cell ability. Thus, the premature senescence of MSCs is a main concern that needs to be addressed to achieve better outcomes in cell therapy.

Cell surface markers were identified as quality-control markers to select functional MSCs, as suggested by previous reports. Our results indicated that the change in EGFR and CD49f protein expression was associated with the cell size of MSCs [28]. Additionally, surface markers, including CD264, CD142, and CD274, have been reported as new markers for isolating MSCs [29–31]. These markers were useful for quality control to characterize stem cell phenotypes destined for therapeutic treatment. Among them, CD264 is a surface marker to select aging MSCs unrelated to the chronological age of the donor; cells expressing this protein exhibit increased senescence-associated *β*-galactosidase (SA *β*-gal) activity and reduced differentiation potential and colony-forming efficiency compared to CD264^–^ MSCs [29, 32]. To confirm that the cell surface proteins on senescent MSCs control the aging process, surface antibody screening was performed. It was previously found that melanoma cell adhesion molecule (MCAM/CD146) among 242 human cell surface markers was downregulated with prolonged in vitro expansion, associated with cellular senescence [27]. Moreover, the surface markers CD47, CD49b, CD274, and EGFR were selectively chosen, showing a significant difference between early and late passages. To validate the association between five surface markers and growth rate, protein levels of these surface markers in UCB-MSCs (early stage, P5) from 10 donors were measured under the same conditions, which could be classified into two groups by the CD47 protein level or growth potential (Group 1 vs. Group 2). Particularly, cells in Group 1 had stopped proliferation soon and observed faster senescence with a lower cumulative PD. Significantly lower levels of CD47 expression were also obtained in Group 1. Overall, the results provided evidence of CD47 as a candidate surface marker for selecting high-growth cells or late senescent cells.

Autophagy plays a key role in maintaining bioenergetic homeostasis through regulating molecular clearance or organelle turnover [33]. Unstable autophagy can lead to cell death or cellular senescence. Autophagy gradually declined cellular modulation with aging, resulting in loss of cellular efficiency [34]. Interestingly, autophagy is necessary for growth and differentiation of MSCs, suggesting that downregulation of autophagy can restrict the therapeutic effect of MSCs [12]. Thus, we hypothesized that autophagy might employ later senescence to control cell growth or senescence processing in MSCs. Senescent cells are identified and characterized by the senescence phenotype, triggering the stable repression of E2F-target genes and repressing some growth-enhancing genes through the recruitment of the retinoblastoma (Rb) tumor suppressor or p38 mitogen-activated [35, 36], as demonstrated by results on protein levels of p38 and Rb. Zhang et al. demonstrated that autophagy plays a protective role in senescence, with autophagy activation before aging having the potential to delay MSC senescence. The ROS/JNK/p38 mechanism pathway plays a key mediating role in autophagy and delayed MSC senescence [37]. Autophagy uses a bulk protein and organelle degradation system and is the main homeostatic cellular recycling process. Three types of autophagy processes included the following: macroautophagy-, microautophagy-, and chaperone-mediated autophagy. Usually, macroautophagy is considered the major form of autophagy. Autophagy is controlled by double-membrane-bound structures called autophagosomes [1, 3, 38].

The autophagic pathway is classified into several phases: initiation, vesicle elongation, maturation, fusion, and degradation [39]. Beclin1, the mammalian ortholog of the yeast protein ATG6, has been known to play a crucial role in the autophagy initiation step [40]. Beclin1 plays a role in the phosphoinositide-3 kinase pathway to activate the formation of autophagic vacuoles [41]. Almost all Atg5 and Atg12 are covalently attached to each other and present as an Atg12-Atg5 conjugate within cells, forming a protein complex in the cytosol [42]. A small fraction of the Atg12-Atg5 complex targets the outer side of the phagophore, which is an intermediate structure during vesicle elongation [43]. LC3B is an autophagosomal ortholog of the yeast protein ATG8 and is a specific marker of autophagosome formation (maturation-fusion-degradation) [44]. LC3BI is localized in the cytoplasm, whereas LC3BII binds to autophagosomes. Autophagy stimulation leads to the conjugation of LC3II [45]. LC3BII binds to autophagosomes and is degraded by lysosomal hydrolases after the fusion of autophagosomes with lysosomes [46]. Recent data has suggested that additional membranes are derived from the Golgi complex, mitochondria, and plasma membrane [47]; however, this process has not yet been admitted. In this study, various autophagy-specific markers (Beclin1, ATG5, ATG12, LC3BI, and LC3BII) were analyzed through the autophagy pathway. Moreover, to further examine the relationship of cellular mechanisms between CD47 expression and autophagy, autophagy-associated proteins were tested in two groups (Group 1 vs. Group 2). These groups showed significant differences in CD47 expression at an early stage (P5), suggesting that growth ability may control autophagic activity. Next, we provided evidence that CD47 expression is significantly inhibited during passaging (from P4 to P13) of MSCs through in vitro aging. Similarly, the autophagy phenotype was markedly reduced in passaged MSCs. To investigate the role of CD47 in autophagy and senescence, it was verified that CD47 siRNA-transfected MSCs exhibited an accelerated senescence phenotype. This data suggests that CD47 is a positive regulator of autophagy processing during cellular senescence.

Moreover, the direct role of CD47 in controlling autophagy activation was demonstrated using a protein array. The 60% of LC3B protein levels, a central protein in the autophagy pathway where it functions in autophagy substrate selection and autophagosome biogenesis, were blocked in CD47 siRNA-transfected MSCs. In addition to ATG3, ATG5, ATG7, ATG13, Beclin1, and LAMP1, autophagy-related proteins revealed 20% suppression of protein levels with CD47 inhibition. Rapamycin, an inducer of autophagy, inhibits the mTOR signaling pathway, which decreases autophagy indirectly by negatively regulating the transcription of genes required for lysosomal function [48]. As expected, rapamycin increased LC3BII expression by inducing autophagy in the scrambled control group. However, results from the CD47 siRNA-transfected group demonstrated that the levels of Beclin1 and LC3BII were activated by rapamycin treatment. To analyze autophagic flux, it is essential to determine the extent to which LC3BII is degraded in a lysosome-dependent manner and how much LC3 puncta are demonstrated with immune staining. The increase in the number of LC3 puncta in the control group in the presence of rapamycin represented the number of autophagosomes, whereas the CD47 siRNA groups revealed fewer LC3B-positive puncta in the presence of rapamycin. These results establish that CD47 is critical for modulating autophagic flux via LC3B-related lysosome regulation. Collectively, this data suggests that CD47 can be a useful candidate marker for predicting the senescence status or good growth of MSCs.

Indeed, results demonstrated that CD47 plays a role in controlling MSC growth, and suppression of CD47 downregulated the senescence process in MSCs. Moreover, CD47 leads to cell death in normal and tumor cells via autophagy. For example, regulation of autophagy by CD47 was also reported in a model of transverse aortic constriction to expose left ventricular heart failure, which indicated that the presence of CD47 could enhance autophagy in injured heart muscle under conditions where TSP1 expression is also increased [49]. Importantly, this study demonstrated that CD47 downregulation resulted in decreased autophagy protein levels. Thus, this data provides new direct evidence that autophagy is a downstream target of CD47 during the development of cellular senescence in MSCs. Here, we observed a novel correlation between CD47 and autophagy in senescent MSCs. To obtain a sufficient number of functional cells, cellular senescence, inevitably induced by long-term culture, has to be evaluated with a therapeutic marker. CD47, as the cell surface marker, can be a useful marker for predicting cellular senescence and applied in potential quality-control assessments for MSC-based therapy. However, further research, which is in progress, is needed to clarify the downstream pathway to understand the positive regulation of autophagy by CD47 during cellular senescence.

## 5. Conclusion

CD47 expression markedly decreased during MSC expansion in vitro, with augmented CD47 downregulation accelerating the senescence phenotype, which affected cell growth. Collectively, this data indicated that CD47 is a key player in autophagy and senescence to maintain and orchestrate MSC growth. Collectively, these results suggest that CD47 is a novel quality-control marker for predicting senescence or selecting good growth in MSCs and could be valuable in quality-control evaluation and for enhancing the therapeutic effect of MSC-based therapy.

## Data Availability

The datasets generated during the current study are available from the corresponding authors on reasonable request.

## Supplementary Materials

Supplementary Materials Table S1: characteristics of MSCs.
