# Supplementary material for: Positively Correlated CD47 Activation and Autophagy in Umbilical Cord Blood-Derived Mesenchymal Stem Cells during Senescence
Source: Stem Cells Int. 2021 Apr 15;2021:5582792. doi: 10.1155/2021/5582792 (PMC8062176; doi:10.1155/2021/5582792)
Supplement: Supplementary Materials — Table S1: characteristics of MSCs. [file 5582792.f1.docx]

**Supplemental Table 1.** Characteristics of MSCs.

| **Group** | **Lot** | **MSC Markers** | | **Osteogenic differentiation** |
| --- | --- | --- | --- | --- |
|  |  | Positive (**≥** 85%) | Negative (≤ 1%) | ALP, Von Kossa |
| Group 1 | MSC 1 | Pass | Pass | Pass |
|  | MSC 2 | Pass | Pass | Pass |
|  | MSC 3 | Pass | Pass | Pass |
|  | MSC 4 | Pass | Pass | Pass |
|  | MSC 5 | Pass | Pass | Pass |
| Group 2 | MSC 6 | Pass | Pass | Pass |
|  | MSC 7 | Pass | Pass | Pass |
|  | MSC 8 | Pass | Pass | Pass |
|  | MSC 9 | Pass | Pass | Pass |
|  | MSC 10 | Pass | Pass | Pass |
